# Inactivated Flagellin-Containing Vaccine Efficacy against Ovine Enzootic Abortion

**DOI:** 10.3390/pathogens13040277

**Published:** 2024-03-24

**Authors:** Maria Kruglova, Nikolai Nikitin, Ekaterina Evtushenko, Irina Matveeva, Aleksandr Mazurov, Igor Pavlenko, Vera Popova, Olesya Bogomolova, Stepan Vasilyev, Evgeniya Markova, Yuri Fedorov

**Affiliations:** 1 All-Russian Scientific Research and Technological Institute of Biological Industry, Biocombinat, 141142 Moscow, Russia; 2 Department of Virology, Lomonosov Moscow State University, 119234 Moscow, Russia

**Keywords:** *Chlamydia abortus*, vaccine, immune response, pregnant sheep, adjuvant, flagellin, protectivity

## Abstract

*Chlamydia abortus* is the etiological agent of abortion and fetal loss in sheep, goats and bovine cattle in many countries. Even though commercially available vaccines can reduce the incidence in sheep, the development of new, safe, and effective vaccines remains high on the agenda. In this study, an evaluation was made of the efficacy of a vaccine candidate, an inactivated antigen based on the extract of outer membrane proteins of a *C. abortus* strain known as *Chlamydia VNITIBP-21*, in combination with recombinant flagellin as an adjuvant. Pregnant sheep (n = 43) were divided into three groups: an experimental vaccinated group, a control infected group and a control non-infected group. The sheep were vaccinated twice, with an interval of 3 weeks, then infected with the homologous virulent strain of *Chlamydia abortus* on pregnancy day 75. The vaccine candidate reduced *C. abortus* shedding in vaginal swabs considerably, in comparison with the control group. In addition, ewes in the experimental group experienced no abortions, while those in the control group experienced instances of abortion, as well as births of weak and nonviable lambs. The findings show that the vaccine candidate proved itself to be promising in combatting the agent of ovine abortion and fetal loss.

## 1. Introduction

*Chlamydia abortus* causes ovine enzootic abortion (OEA). This pathogen of zoonotic potential has been established as the leading cause of reproductive failure in small ruminants and other livestock [1,2]. The disease has a major economic impact, as it is the most common reason for sheep losing their offspring in selected areas of Europe, North America, Africa, and other parts of the world [2].

Infected animals very often manifest no clinical symptoms, but the agent reproduces itself in placenta tissues, causing late abortions or the birth of nonviable or weak lambs [3,4]. The infection has been found to enter healthy sheep and humans through a portal (oral and naval cavities) with bacteria from infected fetal membranes, vaginal excretions or the wool of living or dead lambs, where enormous amounts of bacteria are contained. The agent of this zoonotic infection poses a potential risk for pregnant women, as instances of abortion after contacting pregnant sheep or goats infected with *C. abortus* have been registered among humans [3,4,5].

Countries throughout the world combat this ovine disease by using inactivated vaccines [6,7,8,9] or live attenuated vaccines (Cevac^®^ Chlamydia, Ceva Animal Health Ltd., Wooburn Green, UK; Enzovax^®^, MSD Animal Health, Rahway, NJ, USA; Midiavac, Benchmark Vaccines Ltd., Braintree, UK (licensed by Novartis Animal Vaccines Ltd., Basel, Switzerland); INMEVA^®^ Laboratorios, Hipra SA, Amer, Spain) [10,11,12]. Vaccines of both types have their disadvantages; nevertheless, they reduce incidence rates in comparison with non-vaccinated flocks.

Live or inactivated vaccines against *C. abortus* have been used successfully outside of Russia for many years, but Russia has neither domestic vaccines against OEA nor experience in using imported vaccines to prevent this dangerous disease. The need for an improved strategy for tackling this issue in Russia, including efficient vaccination, is obvious.

Earlier studies have shown that the 40 kDa major outer membrane protein (MOMP) of *C. abortus* is an important target for generating a protective immune response to *C. abortus* infections [13]. The current study aimed to analyze the efficiency of the vaccine, based on the MOMP antigen extracted from inactivated *C. abortus* with recombinant flagellin as an adjuvant, in pregnant sheep experimentally infected with the homologous virulent strain of *C. abortus.*

## 2. Materials and Methods

### 2.1. Animals

A total of 60 primiparous ewes and 10 stud rams of the Romanov breed were procured from an infectious disease-free farm flock in the Mari El Republic, Russia. The animals had no known prior instances of abortive chlamydiosis and had not been vaccinated against chlamydiosis. The sheep were subjected to serological screening for *C. abortus*, using the commercially available PrioCHECK™ Ruminant *Chlamydophila* spp. Ab Kit (ThermoFisher Scientific, Waltham, MA, USA), to measure circulating antibodies to chlamydia, in line with the manufacturer’s guidelines.

In addition, the sheep were administered serological tests prior to the study in order to detect antibodies to *Brucella* spp. (using ID Screen Brucellosis Serum Indirect Multi-species, ID-VET, Grabels, France), *Toxoplasma gondii* (using ID Screen Toxoplasmosis Indirect Multi-species, ID-VET, France), *Coxiella burnetii* (using ID Screen Q Fever Indirect Multi-species, ID-VET, France) and *Maedi-Visna virus* (using ID Screen MVV/CAEV Indirect, ID-VET, France).

Seronegative ewes (n = 60) were randomly distributed among three groups: an experimental group identified as Experimental Group One (n = 30) and two control groups, identified as Control Group Two (n = 15) and Control Group Three (n = 15). Animals in Control Groups Two and Three were used as positive (infected) and negative (non-infected) controls, respectively.

Animals in Experimental Group One were immunized with the vaccine candidate intramuscularly (2.0 mL) twice, with an interval of 21 days. No animals in the control groups were vaccinated.

Animals in all groups were mated with clean-bred, seronegative rams of the Romanov breed for 5 days; then, the ewes were separated from the rams. Pregnancy and progeny viability were diagnosed with ultrasound scanning on day 45 after mating. Non-pregnant animals were removed from the experiment. Pregnancy was eventually diagnosed in 43 ewes, with Experimental Group One being reduced to 23 ewes and the two control groups reduced to 10 ewes each.

**Figure 1:**
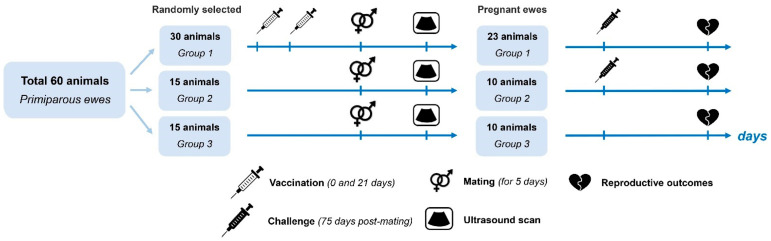
Experimental design for the evaluation of *C. abortus* vaccine candidate efficacy. Group One—experimental group (ewes immunized with the vaccine candidate and infected with the virulent strain), Group Two—control group of non-vaccinated ewes infected with the virulent strain, Group Three—control group of non-vaccinated ewes not infected with the virulent strain.

Experimental Group One (vaccinated) (n = 23) and Control Group Two (non-vaccinated) (n = 10) were infected with the homologous virulent chlamydia strain on day 75. Control Group Three (negative) (n = 10) was not infected and the ewes were kept separate (Figure 1).

### 2.2. Statement of Ethics

This study of vaccine candidate efficacy was carried out in line with Directive 2010/63/EU of the European Parliament and of the Council of 22 September 2010 on the Protection of Animals Used for Scientific Purposes [14], the Guide for the Care and Use of Laboratory Animals (Eighth Edition) [15] and Russian law. The experiments were covered by the Ethics Committee of the Russian Research, Development and Technology Institute of Biological Industry, № 10-01 (13 November 2021).

All ewes and lambs were kept under constant observation for any clinical signs of disease, with the availability of veterinary aid throughout the experiment, including 2 months after lambing.

### 2.3. C. abortus Strain

*Chlamydia VNITIBP-21*, a virulent, abortion-inducing strain of *C. abortus*, was used to prepare the vaccine. The strain was also used to prepare inoculants for the vaccinated group and Control Group Two of pregnant ewes. *Chlamydia VNITIBP-21* is an epizootic virulent strain of *Chlamydia abortus*, isolated from the inner tissues of an aborted sheep fetus at a farm in the village of Turma, Medvedevo District, Mari El Republic, where instances of abortion, stillbirth and the birth of nonviable lambs had been registered. The strain was adapted for replication in chicken embryos and continuous cell culture lines; it was subjected to 25 passages of consecutive culturing in yolk membranes of a chicken embryo [16].

Chicken embryos of 6 to 8 days, from SPF chicken, and the McCoy, ATCC, CRL-1696 cell line (hybrid cell line from human synovial cells and mice fibroblasts) were used in the experiment. The master seed of the *Chlamydia VNITIBP-21* strain was grown in SPF chicken embryos. In summary, infected yolk sacs were ground up using sterile glass beads and dissolved in phosphate buffer saline (PBS). After centrifuging at 5000× *g* for 10 min, the solution obtained was aliquoted and stored in liquid nitrogen. The titer of *C. abortus* elementary bodies in yolk sac material was measured by inoculating decimal dilutions in the McCoy cell culture, as described in [17], after which standardized aliquots were frozen at −80 °C until use. The number of IFUs in McCoy cells was calculated using fluorescence microscopy, with the help of a direct immunofluorescence test using a component kit of Fluorescent Globulin for Chlamydiosis Diagnostics in Animals’ own design (Russian Research, Development and Technology Institute of Biological Industry, Russia). Titring of the inoculate was performed by counting inclusion forming units (IFU) in McCoy cells. The inoculate was diluted in PBS down to 10^6^ IFU/mL.

Master seed material of the *Chlamydia VNITIBP-21* strain was then passaged in McCoy cells, in accordance with previously published recommendations [18,19].

Cells infected with elementary chlamydia bodies were collected using sterile glass balls, 72 h after infection. *C. abortus* elementary bodies were purified by centrifuging with a velocity sedimentation gradient through urografin, as described previously [18]. Purified elementary chlamydia bodies were resuspended in PBS and stored at −20 °C until use.

For infection purposes, freshly unfrozen flasks were diluted with sterile PBS, 0.1 mol, pH 7.2, to final concentrations of 1.5 × 10^6^ IFU/mL. Amounts of 2 mL were injected in ewes subcutaneously, on pregnancy day 75.

### 2.4. Obtaining the Vaccine Antigen

The vaccine antigen from elementary bodies obtained was a complex of outer membrane proteins extracted by a detergent and containing the native major outer membrane protein of chlamydia. The antigen for inclusion in the vaccine candidate was obtained as described in referenced studies [13,20,21,22,23].

In summary, the elementary bodies were incubated in 100 mmol PBS, pH 7.4, containing 10 mmol of EDTA and 2% of sarkosyl (sodium N-lauroylsarcosinate, Sigma-Aldrich, St. Louis, MO, USA), for 1 h at 37 °C, stirred regularly and ultrasonicated (10 s, five times). Then, the mixture was centrifuged at 100,000× *g* for 45 min for sedimentation purposes. The sediment was resuspended and additionally incubated in the same solution containing 10 mmol of dithiothreitol (DTT, Sigma-Aldrich). This mixture was then centrifuged and the resulting sediment of chlamydia outer membrane proteins was suspended in PBS, pH 7.4, and its protein content was measured.

Antigen preparations were stored at −80 °C until vaccine formulation.

The protein in antigen preparation samples was measured at wavelengths of 280 and 205 nm. The major outer membrane protein concentration was also studied, using the Bradford protein assay [24], measuring absorbance at a wavelength of 595 nm. The protein concentration was calculated using a calibration curve plotted with bovine serum albumin (BSA). Protein concentration measurement was carried out using two spectrophotometers: BioMate 3 (Thermo Scientific, Waltham, MA, USA) and Ez Drop 1000 (Blue-Ray Biotech, New Taipei City, Taiwan).

The vaccine candidate was prepared by mixing the major outer membrane protein antigen, containing the final protein concentration of 25 µg, with an adjuvant, using 20 µg per vaccine dose of the commercially available RecFlic-FM adjuvant, as recommended by the adjuvant manufacturer.

### 2.5. Recombinant Flagellin: Profile, Content in the Vaccine

As an adjuvant, recombinant flagellin RecFlic-FM (recombinant flagellin from *Salmonella typhimurium*) was used, (20 µg per vaccine dose).

Recombinant flagellin from *S. typhimurium* was provided courtesy of LLC Firn M, Obolenskoye, Russia. This is a recombinant flagellin protein coded by the fliC gene from Gram-negative *Salmonella typhimurium*. Recombinant flagellin was generated in *Escherichia coli* cells and purified by affinity chromatography; date of manufacture: 15 February 2021; batch number: 5 (LLC Firn M, Obolenskoye, Russia). Identity and purity verification was performed using electrophoretic technology.

### 2.6. Specific Antibody Detection

Blood samples for extracting serum and performing serological tests were taken before vaccination, 14 days after vaccination and subsequently once a week throughout the experiment.

Immunological measurements for circulating specific chlamydial antibodies in blood serum were made using the component kit for chlamydiosis diagnostics via the complement fixation test (or prolonged complement fixation test) of the research team’s own design (Russian Research, Development and Technology Institute of Biological Industry) and the commercially available PrioCHECKTM Ruminant *Chlamydophila* spp. Ab Kit (Thermo Fisher Scientific, USA), in line with the manufacturer’s guidelines.

### 2.7. C. abortus DNA Detection

In order to measure the bacterial load, vaginal swabs were taken from all infected ewes 3 h after delivery. All collected samples (n = 43) were tested with qPCR, as described in the next section.

The presence of chlamydia DNA in ewe vaginal swabs was confirmed by PCR tests, using the commercially available Kylt^®^ *Chlamydia abortus* Reagent Kit, in line with the guidelines of the manufacturer (AniCon, Emstek, Germany) [6]. Ten consecutive dilutions of the *C. abortus* inoculate (10^6^ IFU/mL), for the purpose of plotting the standard curve to calculate the number of *C. abortus* genome copies and isolating the DNA from each sample, were carried out using the Colibri-48 high-performance robotised workstation (fully automated nucleic acid purification from 96 samples, manufactured by LLC NPF Sintol, Moscow, Russia). A DTprime thermal cycler (DNA-Technology, Moscow, Russia) was used for amplification.

### 2.8. Ewe and Lamb Physiological Data Monitoring

The rectal temperature of all animals was measured daily for 5 days after immunization with the vaccine candidate or after infection with the homologous strain of *C. abortus*. The lamb’s body weight was registered at birth and 30 days after birth.

### 2.9. Statistical Analysis

A two-tailed Fisher’s exact test with the Holm–Bonferroni correction was used for comparisons of pregnancy outcome and percentage of viable lambs between groups. A Kruskal–Wallis test was used for comparison of incidence of multifetal and singleton pregnancies between groups. The results of statistical analysis were considered to be significant with a *p*-value of less than 0.05. Statistical processing of the results was carried out using the GraphPadPrism 9.5.1 program (GraphPad Software, La Jolla, San Diego, CA, USA).

## 3. Results

### 3.1. Physiological Status Follow-Up on Ewes after Vaccination

The vaccinated experimental group of animals displayed no clinical signs of reproductive disorder after vaccination, and the behavior of the animals was considered to conform to the physiological norm, as defined from monitoring during the period before virulent chlamydia strain administration. Daily rectal temperatures of vaccinated and control sheep were unchanged after vaccination, and none of the groups displayed higher temperatures. No changes were observed in vaccinated or control animals during pregnancy, from vaccination until ultrasound detection of the pregnancy stage and fetus viability on day 45 after mating. Researchers observed no significant differences from the regular rate of first pregnancy occurrence in this breed of sheep, as 43 of the 60 ewes involved in the experiment fell pregnant after mating.

### 3.2. Detection of Specific Antibodies after Vaccination and Infection with Homologous Strain

**Figure 2:**
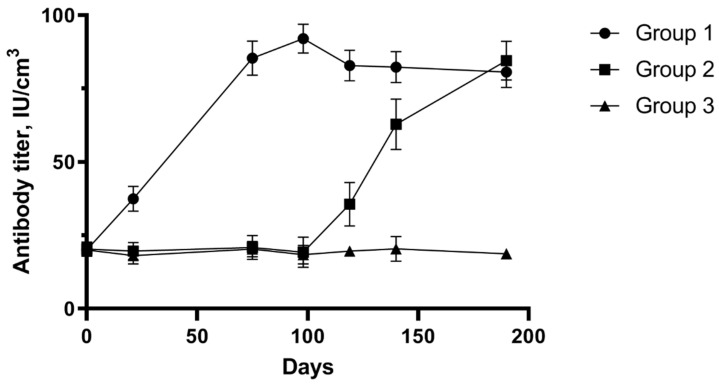
The level of humoral immunity in sheep following immunization with vaccine candidate and challenge with Chlamydia abortus. Blood samples of day 0 correspond to the first day of mating, day 21 to the day of the booster vaccination and day 75 (the 75th day of pregnancy) to infection with the homologous chlamydia strain. Group One—experimental group (ewes immunized with the vaccine candidate and infected with the virulent strain), Group Two—control group of non-vaccinated ewes infected with the virulent strain, Group Three—control group of non-vaccinated ewes not infected with the virulent strain.

Figure 2 displays the results of serological testing of pregnant sheep blood serum after vaccination and infection with the homologous *C. abortus* strain. On the day of vaccination, all animals were seronegative in the ELISA assay. In the sheep of Group One, antibodies were detected by day 14 after vaccination, and their titer increased after the second administration of the vaccine candidate until inoculation of the virulent homologous chlamydia strain. Infecting sheep in the experimental group contributed to antibody production in the animals for 2 weeks, and a constant minor increase was observed throughout the 2 subsequent weeks. The sheep of Control Group Two (non-vaccinated) remained seronegative until administration of the chlamydia inoculate; then, a considerable increase in the number of antibodies was observed, which suggests the development of pathological processes caused by the virulent strain in the animals. Sheep of the experimental group and both control groups lambed 131 to 150 days after the start of the experiment.

The number of chlamydial antibodies showed an upward trajectory in ewes of Control Group Two that experienced an abortion; in contrast, the antibody titer of ewes of Experimental Group One stabilized and was declining gradually by day 190 of the observations.

### 3.3. Impact of Infective Dose of C. abortus Virulent Srtain on Animals

The immunized ewes of Group One were infected on pregnancy day 75 in the same conditions as animals of Control Group Two (unvaccinated), and were monitored for reproductive function disorders caused by *C. abortus*, as well as for the presence of the microorganism in vaginal swabs. None of the ewes in Group One, immunized with the vaccine candidate, experienced an abortion. The only clinical sign observed after the infection was a higher rectal temperature for the initial 48 h. The temperature in non-infected sheep was 39.6 ± 0.1 °C, while the temperature in infected sheep was 40.6 ± 0.5 °C (Group One) and 40.8 ± 0.6 °C (Group Two), 48 h later, respectively.

Observations for each group of ewes after infection with the virulent chlamydial strain are shown in Table 1, which displays the percentage (%) of live lambs, the number of abortions and deliveries with all live lambs, average pregnancy duration, and the average weight of lambs at birth, for each group.

The pregnancy outcome in Control Group Two was statistically significantly different from Group One and Control Group 3 (Table 2). A total of 6 of the 10 ewes in Group Two experienced an abortion, while in the two other groups pregnancy progressed according to the physiological norm. In Control Group Two, multifetal pregnancy resulted in abortions, and three lambs of ewes with a singleton pregnancy were the only lambs to survive. Furthermore, the percentage of viable lambs was higher in the vaccinated group versus the unvaccinated Control Group Two (*p*-value = 0.0003 × 10^−11^). There were no significant differences in the percentage of viable lambs between Group One and Control Group Three (Table 3).

Deliveries of five nonviable lambs, during the lambing period, were registered among the ewes of Group One. They all died 1 to 6 days after birth, but PCR tests of all body samples collected from these lambs were negative to *C. abortus*, proving that *C. abortus* had nothing to do with the perinatal deaths of these lambs. Ewes of Group One had no abortions, and no other instances of reproductive diseases related to *C. abortus* were registered during the studies. Thus, the percentage of reproductive disorders was significantly higher in Control Group Two versus Groups One and Three (Table 2 and Table 3).

The body weight at birth of lambs born from vaccinated ewes of Group One and ewes of Group Three was much higher than that of those born from ewes of Group Two. Pregnancy duration for ewes of Groups One and Three adhered to the physiological norm (see Table 1).

Table 4 summarizes the data on multiple pregnancies, showing that the immunized group of ewes retained the physiological ability to have multiple pregnancies. These data indicate the safety of the vaccine candidate as well as the absence of abortions in vaccinated animals.

**Figure 3:**
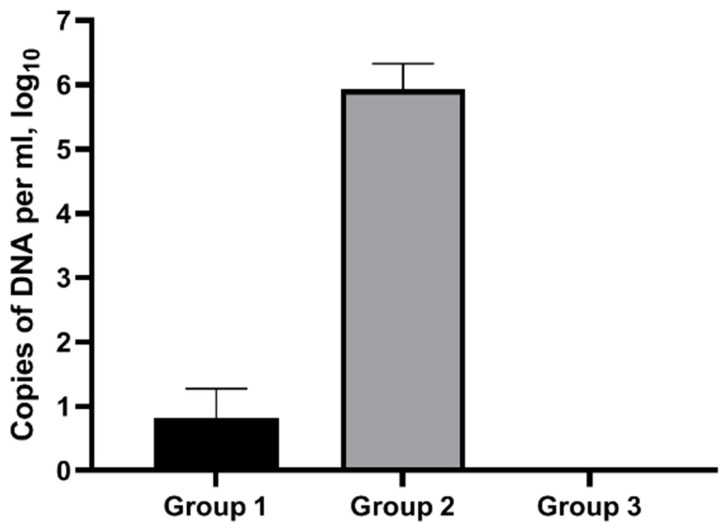
Results of chlamydia genome detection, via PCR, in biological material (vaginal swabs) of ewes following infection with the homologous virulent strain. Group One—experimental group (ewes immunized with the vaccine candidate and infected with the virulent strain), Group Two—control group of non-vaccinated ewes infected with the virulent strain, Group Three—control group of non-vaccinated ewes not infected with the virulent strain.

Figure 3 displays the results of chlamydia DNA detection after delivery. Vaginal swabs taken from ewes of Group One after virulent strain infection contained far fewer *C. abortus* genome copies than those taken from ewes of Control Group Two. It should be noted that chlamydia DNA was detected only in 30.4% of the ewes of Group One. At the same time, the number of *C. abortus* DNA copies in vaginal swabs was high for all animals of Control Group Two (Figure 3).

PCR testing of the material collected from vaginal swabs upon abortion and delivery of Control Group Two showed a high chlamydia content, which certainly suggests the contribution of these microorganisms to the observed pathological processes. This group produced only five live lambs after lambing, and their average weight was 3.45 kg per animal, while the other two groups produced 68 and 35 lambs, for Group One and Group Three, respectively, with an average viable lamb weight of over 4 kg per animal.

The vaccine candidate protected sheep from abortion after infection with the homologous strain. Blood serum samples from all vaccinated sheep contained specific antibodies, which was confirmed using the commercially available ELISA kit.

The data obtained demonstrate the efficacy of the vaccine candidate against infection with the homologous strain. In addition, the studies demonstrated the safety of the vaccine candidate, since no abortions were registered and newborn lambs had a high weight, even though there were a few instances of lamb nonviability.

Studies on the duration of the immunity are continuing into future lambing seasons.

## 4. Discussion

Ewe infection with *C. abortus* usually remains unnoticed until abortion at late pregnancy stages or until the birth of nonviable offspring. This infection has been established as a leading cause of reproductive failure in most sheep-breeding countries worldwide, thus having a major economic impact [2].

Previous studies of vaccine candidate efficacy against OEA caused by *Chlamydia abortus* were focused on live and inactivated vaccines with different ingredients, both recombinant and DNA-based [25,26]. At the same time, attempts at vaccination with a DNA vaccine failed to induce a protective immune response [27,28].

Some countries use commercially available live attenuated vaccines (Enzovax, MSD Animal Health; Cevac Chlamydia, Ceva Animal Health) or inactivated vaccines (e.g., Mydiavac, Benchmark Animal Health). Vaccines of neither type can fully guarantee safety, as chlamydia shedding with birth has been detected, and there is no certainty that the protective immunity duration is estimated correctly [12,29,30,31].

Even though there have been numerous studies on the development of vaccine candidates against *C. abortus*, there remains a shortage of safe and efficient vaccines [6].

Current studies of experimental vaccines are focused on developing next-generation vaccines that will be more efficient, cheaper to manufacture commercially, safer and more stable, and that will not cause illness in animals when used persistently [29,32]. Such vaccines need to incorporate adjuvants among protective antigens of *C. abortus* so that they cause systemic humoral and cell reactions. These requirements emphasize the importance of the ongoing search for a new composition of an inactivated vaccine that will be able to control the disease among pregnant sheep. Production of such a vaccine requires pairing the inactivated antigen with adjuvants to achieve maximum efficacy.

The major outer membrane protein (MOMP) is a crucial immunodominant antigen of *C. abortus* during natural infection, which induces protection when used in a vaccine in the native oligomerous form [13,29,33] in sheep [13], guinea pigs [34], and mice [35,36].

It has been established that MOMP contributes to attachment, by interacting with host cell receptors, as well as to structural integrity maintenance of the bacterium, as chlamydia contains a small amount of peptidoglycan. This protein has a trimeric structure, constitutes about 60% of the total outer membrane protein weight and consists of several conservative T-cell and B-cell epitopes [23,37].

Experimental chlamydia vaccines based on denatured or recombinant MOMP preparations have produced mixed results and provided only partial protection [37,38].

An inactivated *C. abortus* vaccine must primarily aim at inducing a strong cell immune (Th1-like) response, and not only a humoral response. In order to achieve this from the vaccine candidate, a purified antigen based on an extract of outer membrane proteins of the *Chlamydia VNITIBP-21 C. abortus* strain was paired with flagellin, which is recognized by the cell-surface Toll-like receptor 5 (TLR5), contributing to NF-kB activation and subsequent cytokine generation; this makes it a strong T-cell antigen that has potential as a vaccine adjuvant [39].

Studies are underway to develop vaccines with adjuvants that recognize Toll-like receptors [39,40,41]. It is known that the most comprehensive study of the selection of adjuvants was published by Cheng C. et al., in 2011; it featured the efficacy of several ligands for Toll-like receptors (TLR) and the nucleotide-binding oligomerization domain (NOD) [42]. The first adjuvants used in combination with chlamydia antigens were aluminum salts and other adjuvants based on an oil–water emulsion. They did, however, induce the Th2-polarized immune response, which, together with the subsequent discovery of the importance of Th1-polarized responses for chlamydial immunity, prompted researchers to study Th1-polarizing adjuvants. The INMEVA vaccine incorporates a combination of aluminum hydroxide and DEAE dextran as an adjuvant [6].

Earlier research proved that flagellin has a strong adjuvant effect, in many vaccine candidates, against *Yersinia pestis* [43,44], *Plasmodium falciparum* [45], *Clostridium tetani* [46], influenza A virus [47], and West Nile virus [48].

The present study demonstrated that there were no abortions among the experimental group of animals immunized with the vaccine candidate, while there were numerous abortions in Control Group Two, whose animals were not vaccinated. It has been shown that the antigen obtained from elementary bodies of the *C. abortus* strain, with recombinant flagellin as an adjuvant, protects sheep from experimental infection with the virulent homologous strain.

This research also showed that the vaccine candidate against animal chlamydiosis has no negative impact on the reproductive function of pregnant sheep, and that the biological preparation was neither reactogenic nor pyrogenic. The vaccine candidate was made from the antigen of the homologous virulent chlamydia strain isolated from sheep in in Russia. To assess vaccine reactogenicity, immunized animals were monitored for any administration site reaction and for developments in their overall physiological condition. To assess vaccine pyrogenicity, the body temperature of immunized animals was measured systematically, for 14 days after immunization. To monitor whether the vaccine candidate had any negative impact on fetus development, the outcome of lambing and the physiological condition of lambs were assessed.

The experiments performed as part of this study show that the vaccine candidate containing the *C. abortus* major outer membrane protein as an antigen and the adjuvant referred to above can be efficient against ovine enzootic abortion by preventing abortions and mitigating the risk of bacteria shedding during delivery.

An additional experimental requirement is to obtain more data on the level and duration of colostral immunity, through the serological testing of blood serum from lambs born to immunized pregnant sheep. In addition, further studies are necessary to establish the extent of the persistence of immunity into future lambing seasons, and to evaluate the cell and humoral immune response after the infection of vaccinated sheep with heterologous chlamydia strains isolated in Russia.

## Figures and Tables

**Table 1:** Vaccine candidate efficacy assessment based on the number of reproductive disorders caused by *C. abortus* in ewes. Group One—experimental group (ewes immunized with the vaccine candidate and infected with the virulent strain), Group Two—control group of non-vaccinated ewes infected with the virulent strain, Group Three—control group of non-vaccinated ewes not infected with the virulent strain.

	Group 1	Group 2	Group 3
Number of ewes	23	10	10
Percentage of live lambs, %	93.1	15.2	94.6
Total lambs (viable lambs)	73 (68)	33 (5)	37 (35)
Abortions	0	6	0
Number of deliveries/deliveries with all live lambs	23/21	4/3	10/9
Early deliveries	2	3	0
Number of non-viable or stillborn lambs	5	28	2
Lamb’s weight at birth	4.1 ± 0.54	3.45 ± 0.34	4.4 ± 0.46
Pregnancy duration	140.5 ± 5.7	131.1 ± 9.8	145.2 ± 5.2

**Table 2:** Statistical analysis of the differences in pregnancy outcome (delivery/abortion) between groups. For comparison, the two-tailed Fisher’s exact test was used. The Holm–Bonferroni method was applied for adjusting *p*-values for multiple comparisons. *p*-values between groups with statistically significant differences are highlighted in green. *p*-values between groups without statistically significant differences are highlighted in orange.

Group of Ewes	Group 1	Group 3
Group 2	0.0006	0.022
Group 1		1

**Table 3:** Statistical analysis of the differences in percentage of viable lambs. For comparison, the two-tailed Fisher’s exact test was used. The Holm–Bonferroni method was applied for adjusting *p*-values for multiple comparisons. *p*-values between groups with statistically significant differences are highlighted in green. *p*-values between groups without statistically significant differences are highlighted in orange.

Group of Ewes	Group 1	Group 3
Group 2	0.0003 × 10^−11^	0.0006 × 10^−8^
Group 1		1

**Table 4:** The number of multifetal pregnancies in ewes. Group One—experimental group (ewes immunized with the vaccine candidate and infected with the virulent strain), Group Two—control group of non-vaccinated ewes infected with the virulent strain, Group Three—control group of non-vaccinated ewes not infected with the virulent strain. There were no statistically significant differences between the groups (*p*-value = 0.7833, Kruskal–Wallis test).

Number of Fetuses	Number of Animals		
	Group 1	Group 2	Group 3
1	5	3	1
2	6	1	3
3	4	2	1
4	0	0	0
5	4	2	3
6	4	2	2
Total	73	33	37

## Data Availability

Data are contained within the article.
